# Evaluation of collapsibility characteristics in deep loess strata using in-situ sand well immersion test

**DOI:** 10.1038/s41598-026-53514-3

**Published:** 2026-05-21

**Authors:** Gengsheng Yan, Zhenming Lu, Xiangyang Hu, Ming Zhang, Dengli Pan

**Affiliations:** 1 Northwest Engineering Corporation Limited, PowerChina, Xi’an, China; 2 Zhengzhou University of Aeronautics, Zhengzhou, China

**Keywords:** Loess, Collapse, In-situ sand well immersion test, Laboratory test, Engineering, Environmental sciences, Natural hazards, Solid Earth sciences

## Abstract

The significant collapse settlement of deep loess upon water infiltration poses a serious threat to engineering construction safety. Accurate evaluation of collapsibility characteristics is a critical issue in geotechnical engineering. Evaluation methods relying on laboratory tests often diverge from field results. This study focuses on the Shenheyuan loess. It conducts in-situ sand well immersion tests to monitor water migration in the loess stratum and settlement of soil layers at various burial depths during immersion. The results are compared with laboratory collapse test data. The findings indicate that during the in-situ sand well immersion test, the monitored settlements at all points were small, well below the 70 mm criterion for self-weight collapsibility in loess. Therefore, the site is classified as a non-self-weight-collapsible site. In contrast, laboratory tests classify the site as self-weight collapsible with a collapsibility level of grade II. A comparative analysis with the adjacent site confirms that evaluating collapsibility characteristics using an in-situ sand well immersion test is more reliable. The discrepancy between the laboratory test and the in-situ sand well immersion test primarily stems from the regional correction coefficient specified in the code, which fails to account for site-specific factors, such as high groundwater levels, climatic conditions, and soils with high clay content. Finally, a preliminary site-specific correction coefficient of 0.03 is back-calculated based on the measured field response. This study demonstrates the effectiveness of the in-situ sand well immersion test for evaluating the collapsibility of deep loess. It provides an important reference for geotechnical design under similar special geological conditions.

## Introduction

Collapsible loess is a type of soil with high strength in its natural state. When water saturates it, the macroporous structure quickly collapses and cementation weakens. This causes significant extra deformation^1,2^. Such soils are common in arid and semi-arid mid-latitude regions worldwide, especially on China’s Loess Plateau. Its sudden and uneven collapse often leads to severe engineering hazards. These include uneven building settlement^3–5^, ground fissures^6–8^, and underground pipeline ruptures^9,10^, threatening long-term infrastructure safety.

Evaluation of loess collapsibility primarily relies on two methods: laboratory compression tests and in-situ large-scale immersion tests^11–14^. Engineering practice shows that there are discrepancies between the self-weight collapse settlement calculated from laboratory tests according to the code for building construction in collapsible loess regions (GB 50,025–2018)^15^ and in-situ-measured values^16–18^. These differences are mainly attributed to factors at three levels: testing conditions^19^, soil structure^20^, and calculation theory^21^. In-situ sand well immersion tests are considered a more accurate method for evaluating site collapsibility types. Yang et al.^22^, Su et al.^23^, Xu et al.^24^, Qu et al.^25^, and An et al.^26^ conducted field immersion tests to investigate the collapse deformation characteristics of loess strata. For loess sites where in-situ sand well immersion tests are not feasible, Wang et al.^27^ proposed a new method considering the initial structure of loess to evaluate collapsibility, as there is a specific correlation between the collapsibility coefficient and basic physical indices of loess^28^. This method yielded results closer to field tests than laboratory tests. However, for evaluating the collapsibility characteristics of deep loess, the reliability of the aforementioned measurement methods remains insufficient. In recent years, in-situ sand well immersion tests have been developed to measure settlement deformation of loess strata at specific depths under water saturation^2,4,5^.

Therefore, this study takes the Shenheyuan loess as the research subject, conducts in-situ sand well immersion tests to monitor water migration and the deformation responses of loess layers at different depths, and compares the collapse results with laboratory test results. The aim is to provide a basis for accurate evaluation of loess foundation collapsibility characteristics.

## Test Site

**Fig. 1:**
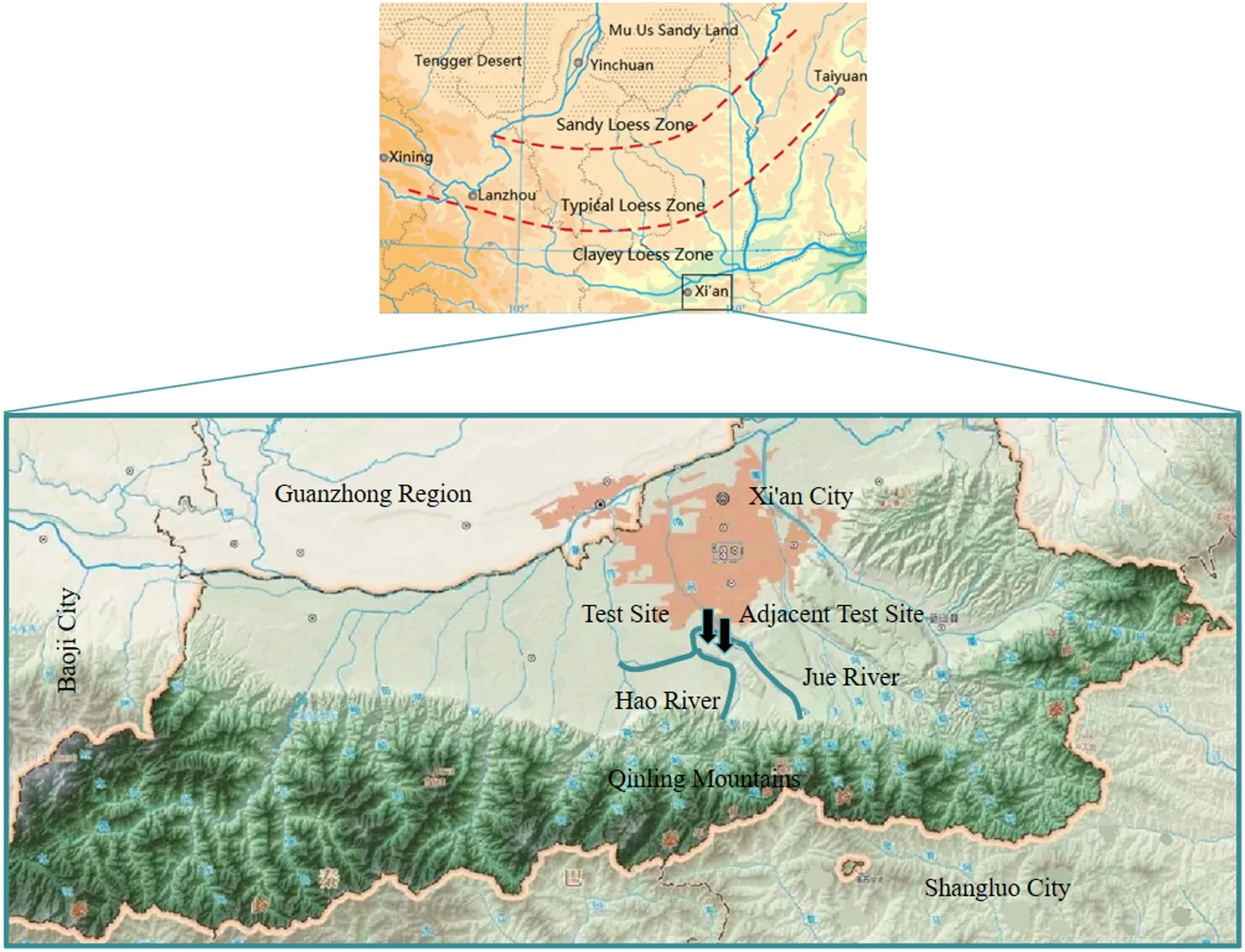
Location map of the test site.

The test site is situated at the southern edge of the Chinese Loess Plateau (Fig. 1). With a ground elevation of approximately 473 m, the area features relatively flat topography. Geomorphologically, it belongs to a first-level loess tableland, known as the Shenheyuan Loess Tableland. Regarding hydrogeological conditions, the groundwater table depth at the test site ranges from 30 to 33 m, which corresponds to the stable groundwater level measured during the site investigation and falls within the site’s long-term annual fluctuation range. Finally, the stratum within the upper 25 m depth at the test site is presented in Table 1.

**Table 1:** Lithology of the test site.

Stratum	Depth (m)	Soil type
Holocene anthropogenic fill (layer I)	0 ~ 1.0	Backfill soil
Upper pleistocene aeolian deposit (Layer II)	1.0 ~ 9.5	Loess
Upper pleistocene eluvial deposit (Layer III)	9.5 ~ 12.6	Paleosol
Middle pleistocene aeolian deposit (Layer IV)	12.6 ~ 22.8	Loess
Middle pleistocene eluvial deposit (Layer V)	22.8 ~ 25.0	Paleosol

## Test scheme

### Layout of settlement observation benchmarks and water content sensors

**Fig. 2:**
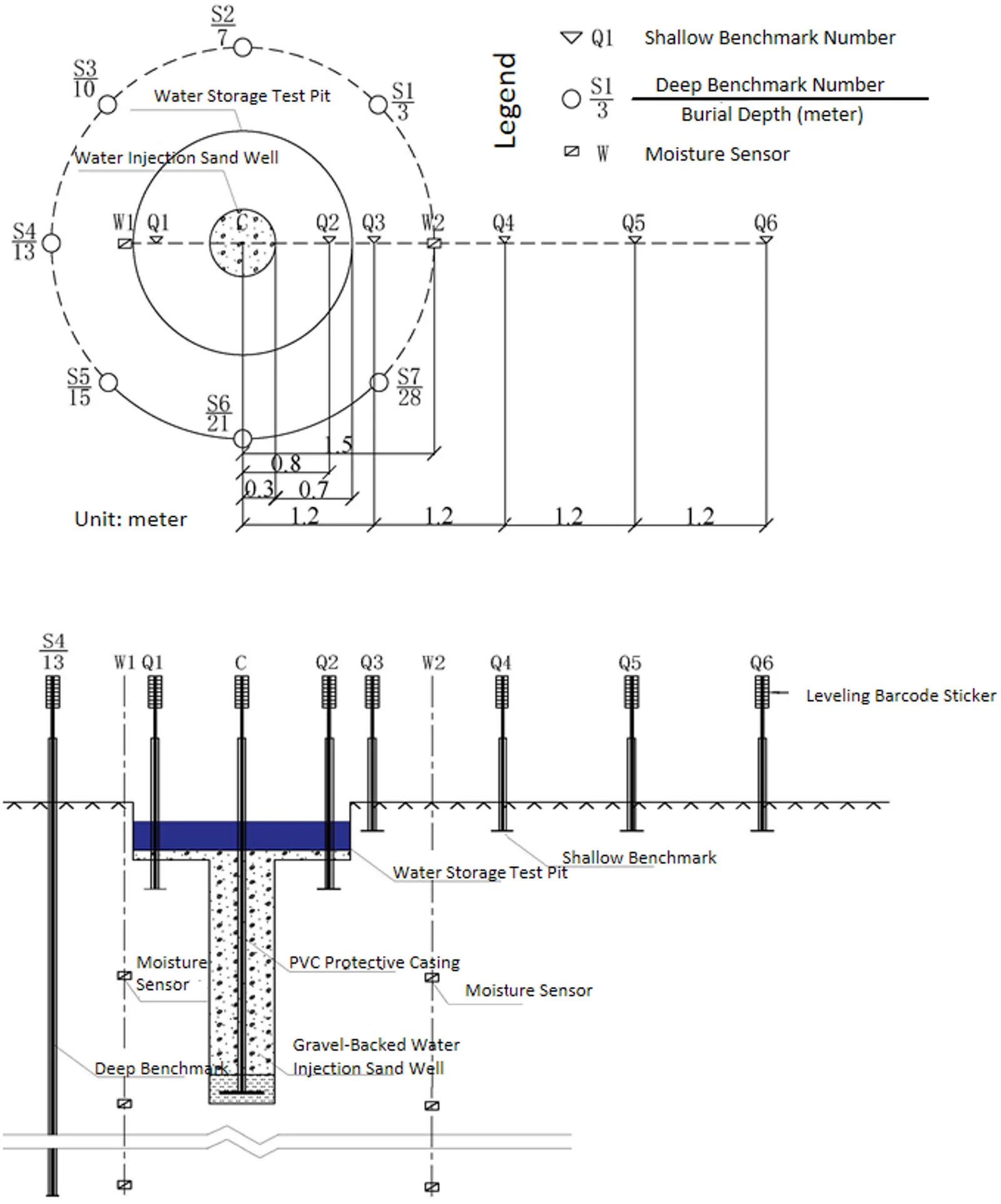
Layout of settlement observation benchmarks and water content sensors.

The sand well was excavated to a depth of 24 m. As a result, the central bearing plate was embedded at that depth, penetrating the loess layer. Figure 2 shows the layout of the settlement observation benchmarks. Fourteen settlement observation benchmarks were installed: 7 deep, 6 shallow, and 1 central. The deep benchmarks, numbered S1 to S7, are at depths of 3, 7, 10, 13, 15, 21, and 28 m. They are uniformly distributed on a circumference with a radius of 1.5 m from the sand well center. The shallow benchmarks, numbered Q1 to Q6, are all embedded at a depth of 0.5 m. The central benchmark is numbered C. Two groups of water content sensors, W1 and W2, were installed. The W1 group is at the edge of the test pit, 1.0 m from the sand well center, with sensors at depths of 5, 8.5, 14.5, and 17 m. The W2 group consists of six sensors placed 1.5 m from the sand well center. Their depths are 3, 5, 15, 19, 21, and 28 m, respectively.

### Design of the in-situ sand well immersion test scheme

**Fig. 3:**
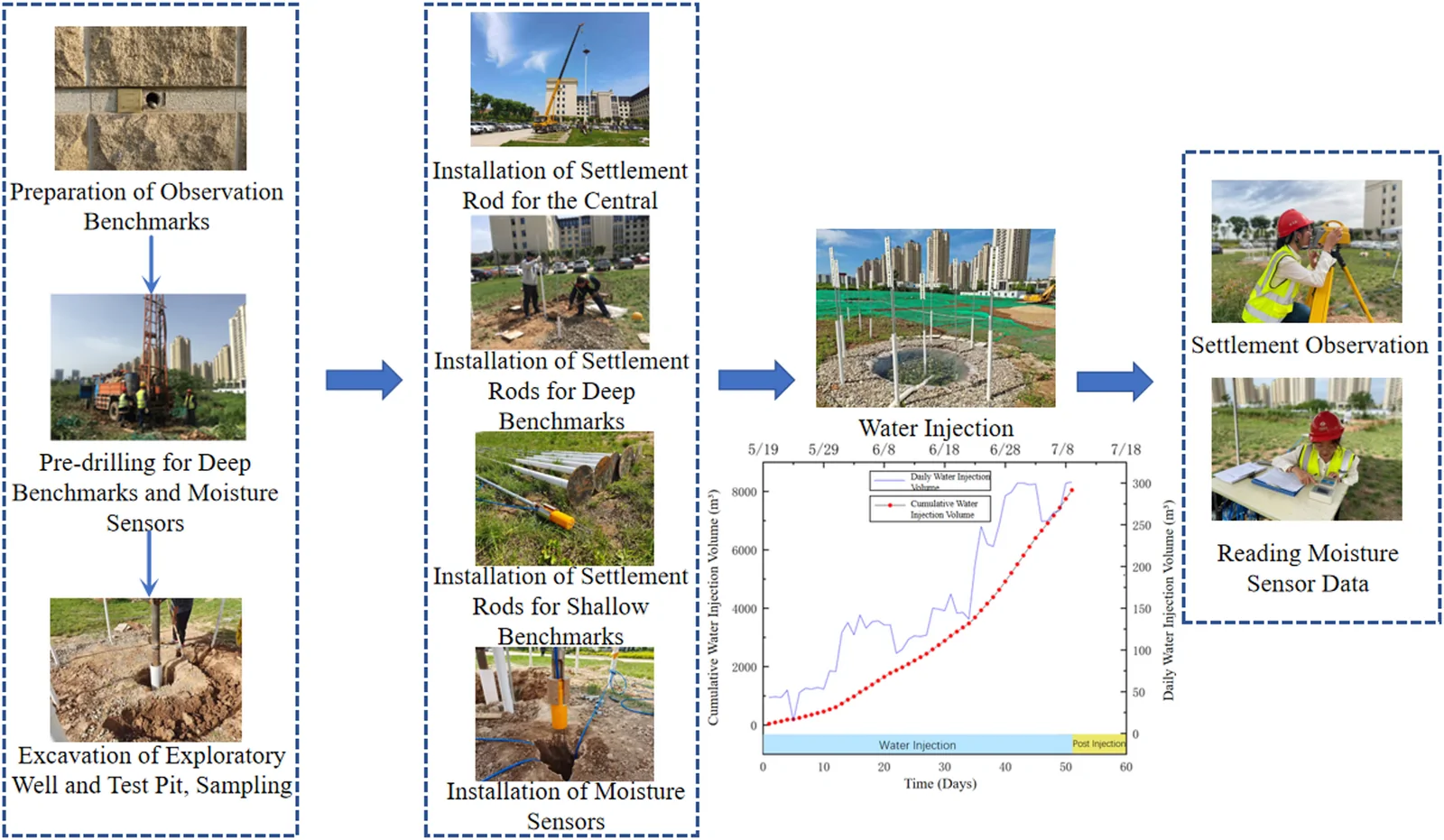
Workflow of the in-situ sand well immersion test.

The workflow of the in-situ sand well immersion test is shown in Fig. 3. Initially, pre-drilling is conducted at the designated burial depths for the deep benchmarks and water content sensors. Next, an exploratory well (i.e., the sand well before gravel backfilling, consistently referred to as the sand well hereafter) with a diameter of 0.5 m and a depth of 24 m is excavated. Subsequently, centered on this well, a test pit with a diameter of 2.0 m and a depth of 0.8 m is excavated. Following this, the excavated soil in a loose condition at the bottom of the sand well is removed, after which a 10 cm-thick permeable sand cushion layer is laid and compacted. Then, a prefabricated settlement-rod assembly is installed in the exploratory well. The bottom of this assembly is fixed to a circular bearing plate, and the rod is sleeved with a polyvinyl chloride (PVC) protective casing to ensure it is laterally unconfined and can move freely up and down with the deformation of the loess layer. The well is then initially backfilled uniformly with a 20-cm-thick layer of medium-fine sand; this step also helps minimize deformation of the bottom soil and the bearing plate caused by the impact of falling gravel during subsequent backfilling. Finally, the well is backfilled with gravel (20–40 mm particle size) to the ground surface. Meanwhile, a settlement rod is welded to one corner of the bearing plate. A steel plate is welded to the top of this rod, and a leveling barcode sticker is affixed to the steel plate. For both shallow and deep benchmarks, the settlement plates are welded to their respective rods. These assemblies are then placed in pre-prepared installations: shallow benchmarks in 40-cm-diameter, 50-cm-deep circular pits, and deep benchmarks in their pre-drilled holes. The rods protrude 2.0 m above the ground surface and are sleeved with PVC protective casings. Next, the surrounding soil is backfilled and compacted in layers. The water content sensors are installed, backfilled, and compacted in layers with plain soil. After completing installation, the sensor cables are routed to a point 20 m away from the center of the sand well.

Following the completion of sand well test preparations, continuous monitoring of all settlement benchmarks and water content sensors is conducted. Settlement readings were manually recorded twice daily (at 08:00 and 18:00) during the active injection period. Water immersion begins only after readings have fully stabilized, defined as a vertical fluctuation of less than 0.1 mm/day over 72 h. Throughout the immersion process, the target water head in the test pit was maintained at approximately 80 cm using a staged water supply, real-time field adjustments, and supplementary manual replenishment when necessary. During the initial filling stage, water was introduced over a wooden baffle plate to dissipate kinetic energy and prevent hydraulic scouring of the natural loess surface at the bottom of the test pit. According to the Code for Building Construction in Collapsible Loess Regions (GB 50,025–2018), monitoring continues for at least 10 days after water immersion stops, and the test is terminated only when the average daily settlement over 5 consecutive days does not exceed 1 mm/d. The water immersion test was conducted from May 16 to July 18, 2021, for a total of 64 days, and was terminated at that time because the average daily settlement over five consecutive days had decreased to below the code-specified threshold of 1 mm/d. This period included 3 days of initial baseline monitoring before immersion, 51 days of active immersion into the test pit, and 10 days of post-immersion monitoring. A total of 8044 m^3^ of water was injected into the sand well during the test, resulting in an average daily injection volume of approximately 157.7 m^3^. The maximum daily water injection volume of 301 m^3^ occurred on July 8, while the minimum daily volume of 15 m^3^ was recorded on May 24, 2021. The temperature of the injected water was regularly monitored and maintained relatively constant (approximately 20 ± 5 ℃) to minimize temperature-induced viscosity variations in the seepage field.

As a large-scale in-situ engineering test, the present in-situ sand well immersion test was conducted at a single test pit and did not include strict parallel repetitions because of practical constraints related to site conditions, construction organization, schedule, and cost. Therefore, the representativeness of the present results mainly relies on the multi-point, multi-depth monitoring layout, the relatively long observation period, and the consistency with nearby-site immersion evidence, rather than on conventional replicate testing.

## Test results and analysis

### Variation in water content of the loess stratum during water immersion

**Fig. 4:**
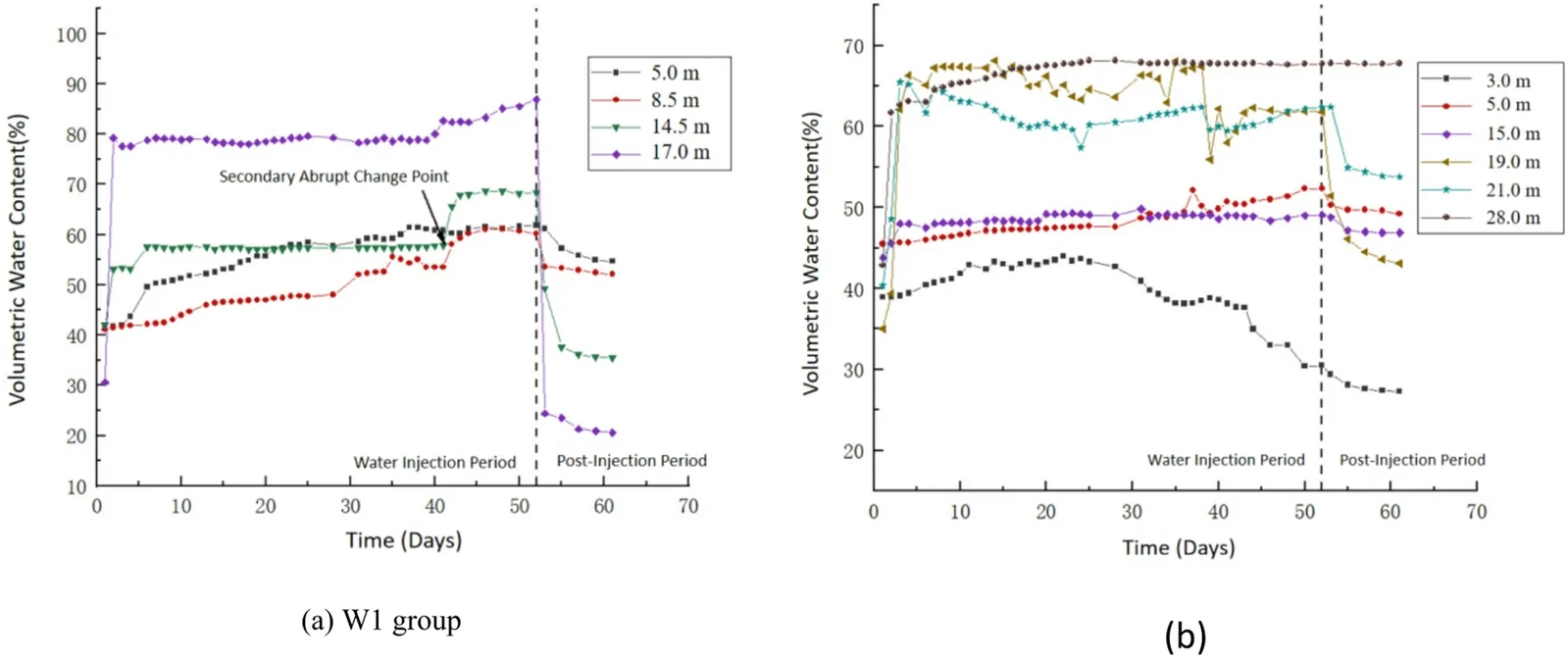
The water content over time at different locations of loess stratum during the in-situ sand well immersion test.

The water content at different locations in the loess stratum during the in-situ sand well immersion test is shown in Fig. 4. For the W1 group (Fig. 4a), significant and abrupt changes in water content are observed at depths of 17.0 m and 14.5 m on the 2nd day of immersion. The change at a depth of 5.0 m occurs on the 6th day. In contrast, at 8.5 m depth, water content increases gradually during the early stages of immersion. An abrupt change in the water content vs. time curve indicates the arrival of the wetting front at that sensor location. Thus, deeper sensors detect changes before shallower ones, indicating that the wetting front first reaches deeper soil layers and then moves upward. This occurs because the voids between the gravel in the well form a direct water channel to the sand well bottom during the test. The stratum below the sand well base is wetted first. As the water level in the sand well rises, the depth of the loess layer affected by water content decreases. Changes in water content then occur in the shallow soil. In Fig. 4a, secondary abrupt changes in water content appear at depths of 8.5 m, 14.5 m, and 17 m on the 41st day. This results from the change in water supply method, shifting from intermittent tank supply to continuous hose supply. As a result, the water head in the test pit rises from an intermittent state to a steady height of 80 cm. This shows that water head pressure influences moisture diffusion. A higher, sustained water head pressure leads to more complete soil saturation.

The pattern of water content variation for the W2 group (Fig. 4b) mirrors that of the W1 group: deeper sensors detect changes earlier. Unlike the four deeper sensors, the 5 m sensor shows a gradual increase after detecting moisture, indicating proximity to the wetting front. The 3 m sensor is unique: after a slight early-stage increase, its readings decline in the mid to late stages. Installed at a shallow depth above the wetting front, it initially balances evaporation loss and moisture supply, resulting in stable readings. However, the shallow, porous soil and the onset of hot summer weather increase evaporation, causing a continuous decrease in readings.

**Fig. 5:**
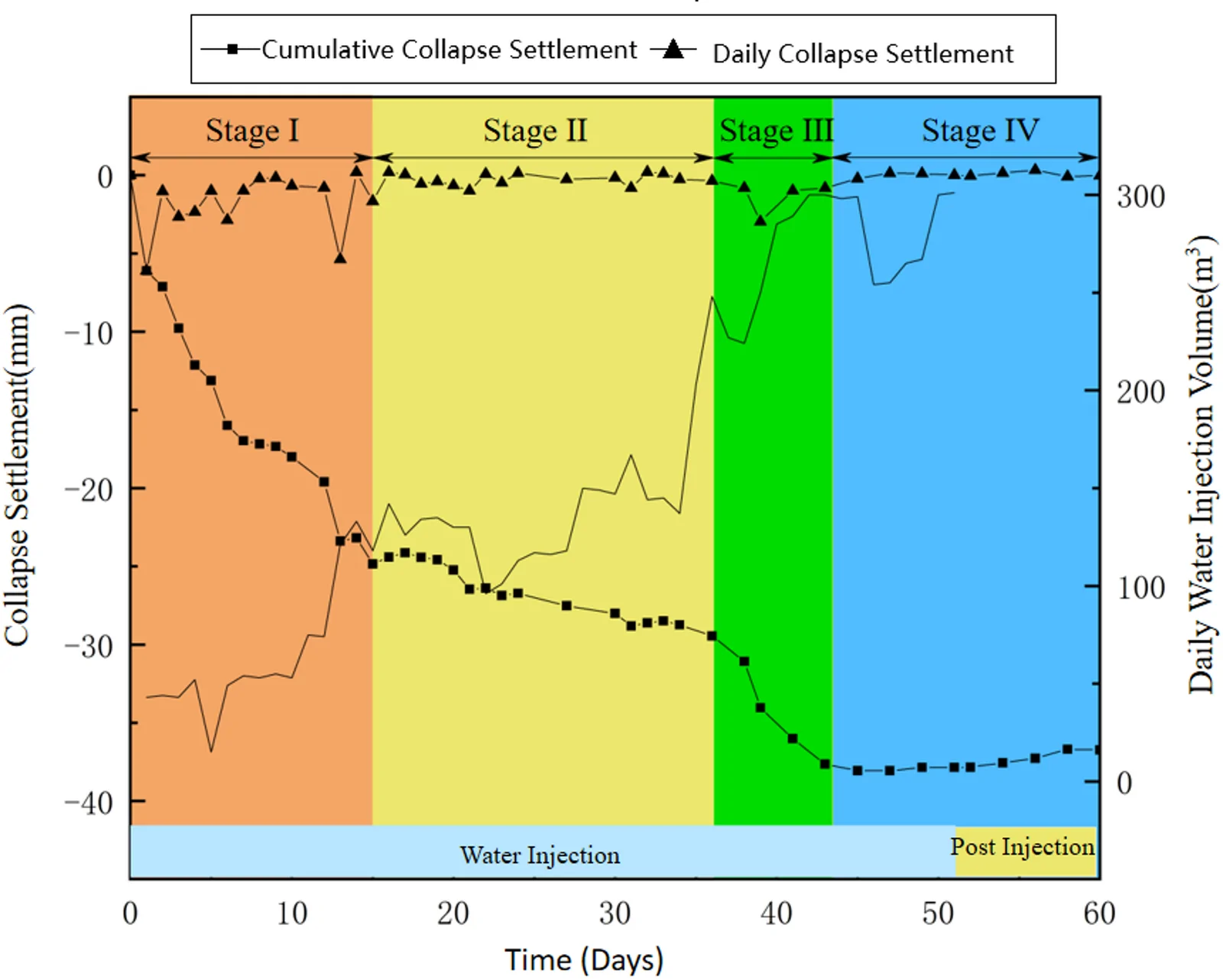
Curves of collapse deformation versus time for the central benchmark.

**Fig. 6:**
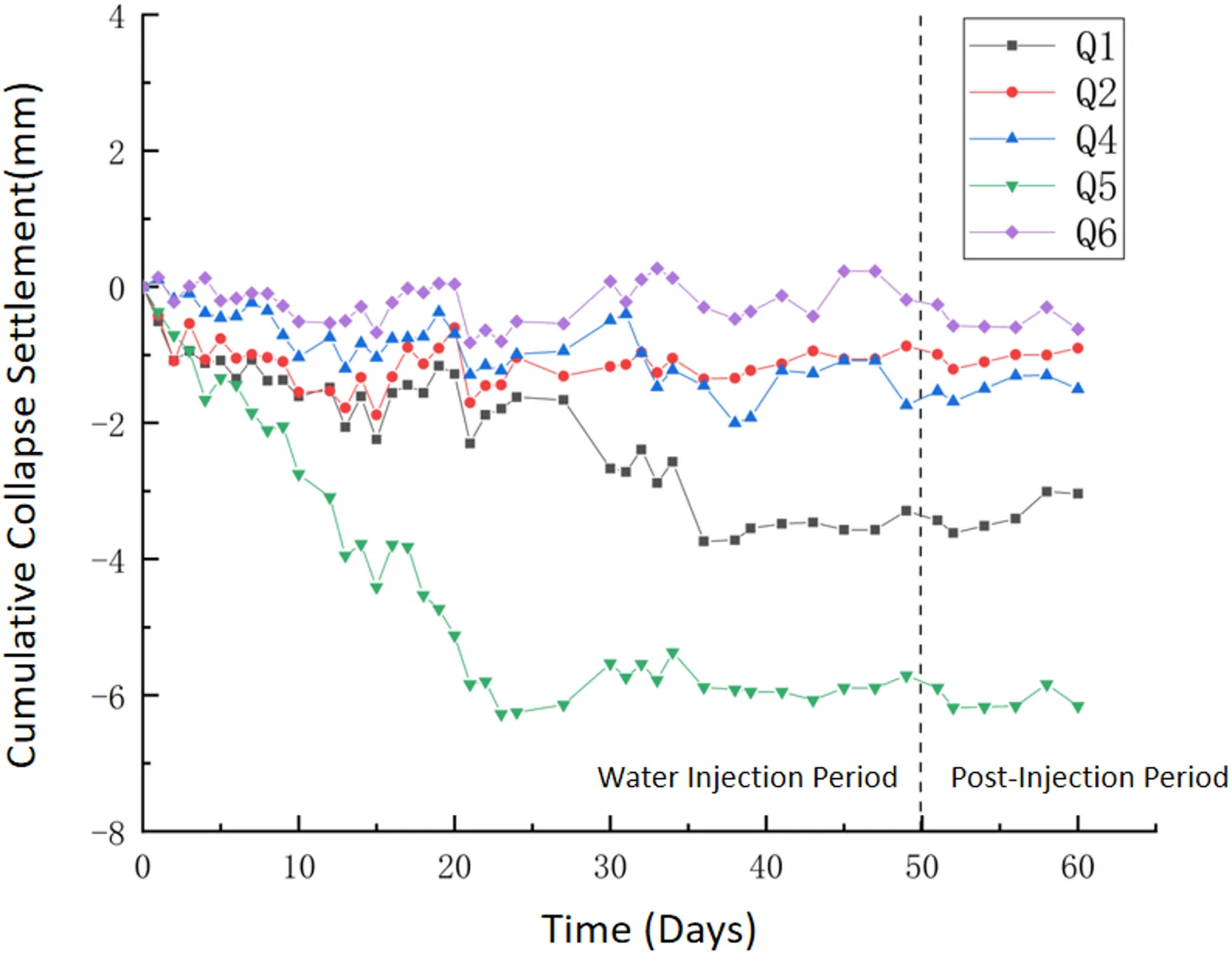
Curves of collapse deformation versus time for the shallow benchmarks.

**Fig. 7:**
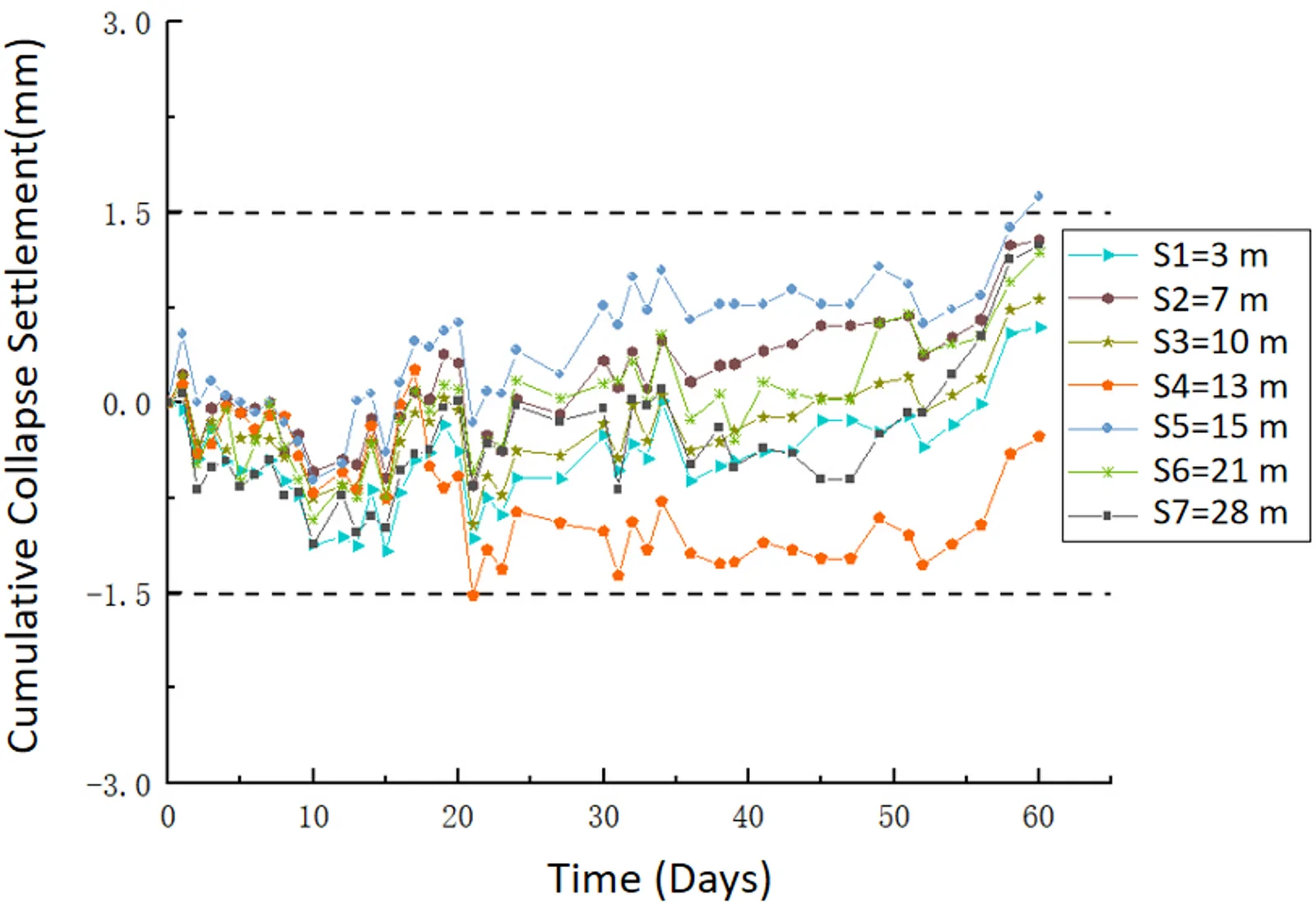
Curves of collapse deformation versus time for the deep benchmarks.

The pattern of water content variation in the loess stratum during the immersion process is influenced by multiple factors, such as burial depth, water head pressure, and distance from the sand well center. In general, when the wetting front reaches the installation depth of a water content sensor, the measured value increases sharply over a short period. After reaching its peak, the value fluctuates slightly around the saturation value. After Water immersion, the sensor readings decrease to varying degrees and subsequently stabilize at a relatively constant value. It should be noted that Figs. 4, 5, 6, 7 present the original time-history records of individual monitoring points from a single large-scale in-situ test, rather than averaged results from repeated parallel tests; therefore, conventional statistical error bars are not directly applicable to these figures.

### Collapse deformation of the loess stratum during water immersion

- (1) Collapse deformation of the central benchmark

Figure 5 presents the curve of collapse deformation versus time for the central benchmark C of the sand well, including the total collapse amount, the daily collapse amount, and the daily water injection volume. Calculations indicate that the total self-weight collapse of the soil layer below the base of the sand well is 38 mm. Based on the settlement rate of the central benchmark C (shown in Fig. 5), the settlement process is divided into four stages:

① Rapid collapse stage (days 0–15). Water is routed directly to the base of the sand well through voids among the gravels, resulting in abrupt shifts in the settlement benchmark. As the water table in the sand well rises, the macropores beneath the well base gradually saturate, triggering intense collapse. The peak daily collapse during this period reaches 6.1 mm. Although the daily water inflow is limited, the curve for this stage remains steep, signifying a high collapse rate and pronounced daily settlement.
② Slow collapse stage (days 16–36). After rapid collapse deformation, the medium- and small-sized pores in the soil also become progressively saturated as the daily water injection volume increases. The soil reaches a higher degree of saturation, and collapse deformation develops slowly, resulting in relatively small daily settlement.
③ Water head induced collapse stage (days 37–44). The daily water injection volume continues to increase. The water head pressure increases as the water level in the test pit rises, and the depth of water diffusion from the well into the underlying strata increases, too. Collapse settlement continues to develop, increasing cumulative collapse.
④ After the water head induced collapse stage, the collapse stabilization stage (days 45–60) is observed. The average daily settlement is less than 1.0 mm/d, indicating that settlement deformation has stabilized.

- (2) Collapse deformation at shallow benchmarks

Figure 6 illustrates the time-dependent collapse deformation for the shallow benchmarks. Q3 was omitted due to its proximity to Q2 and W2. As indicated in Fig. 6, deformation at Q2, Q4, and Q6 remains negligible during both immersion and post-immersion periods, with final settlements not exceeding 1.0 mm at test completion. In contrast, Q1 and Q5 exhibit greater settlements of 3.5 mm and 6.2 mm, respectively. Q1 and Q2, both located in the test pit and equidistant from the sand well center, display markedly different post-immersion settlements, highlighting spatial variability in shallow deformation within the test pit. Q5, situated 3.6 m from the sand well center, shows the largest settlement, indicating that the wetting effect extends at least this far under current conditions. However, due to spatial limitations of the monitoring layout, the data cannot quantitatively distinguish among local stratigraphic variation, uneven moisture redistribution, or boundary effects. Therefore, the present observations only support a minimum verified wetting-influence radius of 3.6 m under the current test conditions, whereas the extent of wetting or saturation beyond the monitored area remains uncertain and should not be interpreted as fully constrained by the present monitoring arrangement. Additionally, daily deformation at all benchmarks remains below 1 mm, indicating minimal variation.

- (3) Collapse deformation at deep benchmarks

Figure 7 presents the curves of collapse deformation over time for the deep benchmarks during water immersion. As shown in Fig. 7, the cumulative collapse deformation of all 7 deep benchmarks is minimal in the test, fluctuating between − 1.5 mm and 1.5 mm. Most benchmarks, apart from S4, generally exhibit an upward trend. S4 is at a soil depth of 13 m, within the Middle Pleistocene loess, and shows relatively greater collapsibility. Therefore, S4 displays slight settlement deformation. The upward trends at several deep benchmarks may be due to the restraining effect of interbedded paleosol layers. Possible local swelling upon wetting has also been suggested by previous studies on loess microstructural evolution and hydro-mechanical behavior^18,21,29–32^. However, this interpretation should be regarded as tentative since no dedicated swelling test or direct verification was conducted in the present study. Deep collapse deformation reflects the collapse of soil layers at various depths below the ground surface. It has significant value for evaluating the lower limit depth of self-weight collapse, selecting foundation embedment depths, and determining geotechnical design parameters.

The monitoring results from all benchmarks indicate that no significant collapse deformation has occurred in the loess stratum. The maximum self-weight collapse measured is 6.5 mm, which is less than the 70 mm criterion. Based on the results of the in-situ sand well immersion test, the loess can therefore be classified as a non-self-weight-collapsible site.

## Discussion on collapsibility evaluation methods

### Comparison of collapsibility evaluation methods

In this study, 56 undisturbed samples were collected from the test site (at sampling points SJ03, TJ02, and TJ10) for laboratory compression tests. The boreholes SJ03, TJ02, and TJ10 used for laboratory comparison were all located within a 20 m radius of the central sand well. The tests determined the maximum burial depth of collapsible soil layers to be 22 m. Soil layers with a self-weight collapsibility coefficient δzs > 0.015 are primarily distributed in the loess layer③, where the maximum δzs value reaches 0.048. The calculated self-weight collapse settlement, measured from the ground surface and using the regional correction coefficient β₀ = 0.9 recommended by the loess code^15^, ranges from 176 to 255 mm. The total collapse settlement, measured from the minimum excavation depth of 6.1 m (the designed foundation embedment depth for the proposed structures), ranges from 199 to 309 mm. Based on these results, the test site is classified as self-weight collapsible, with a foundation collapse grade of II (moderate). The calculation results are summarized in Table 2.

**Table 2:** Calculated results of collapse based on laboratory test.

Exploratory well number	Self-weight collapse settlement/mm	Criterion/mm	Assessment Result
SJ03	210	> 70	Self-weight collapsible
TJ02	255	> 70	Self-weight collapsible
TJ10	176	> 70	Self-weight collapsible
Exploratory well number	Calculation depth range/m	Total collapse settlement/mm	Assessment result
SJ03	6.1 ~ 22.0	199	Ⅱ(Moderate)
TJ02	6.1 ~ 22.0	309	Ⅱ(Moderate)
TJ10	6.1 ~ 22.0	247	Ⅱ(Moderate)

**Fig. 8:**
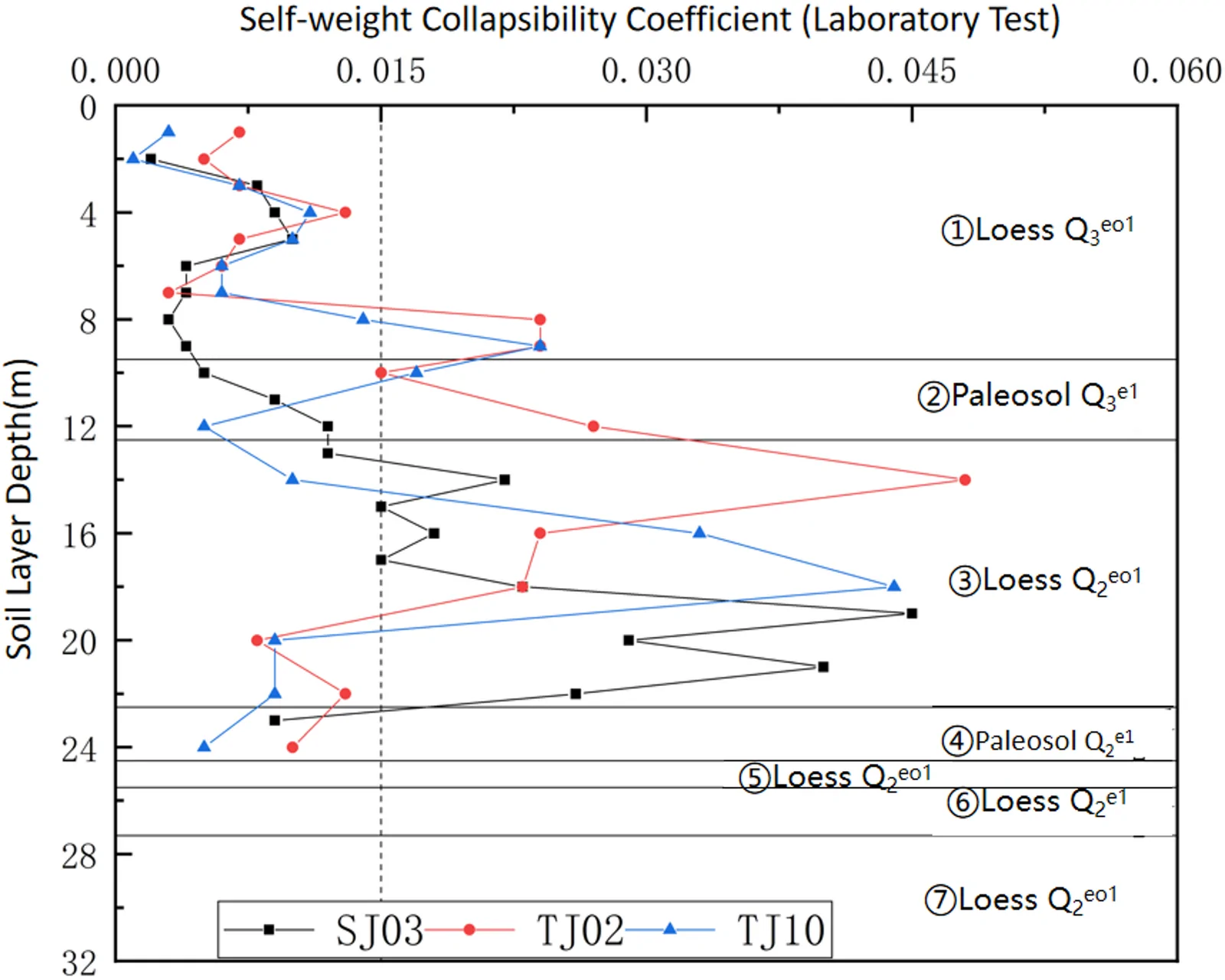
The curve between the self-weight collapsibility coefficient and depth for the loess.

Figure 8 illustrates the relationship between the self-weight collapsibility coefficient and depth for loess at the test site, derived from laboratory tests. As shown in Fig. 8, only Layer ③ (Middle Pleistocene Q₂) possesses a self-weight collapsibility coefficient exceeding 0.015. The self-weight collapsibility coefficients of other soil layers are consistently below 0.015. This confirms that only Layer ③ constitutes self-weight collapsible loess. It is predominantly distributed between 12.2 and 22.5 m in depth. This clarifies why benchmark S4 (13 m depth) experienced slight settlement during the in-situ sand well immersion test shown in Fig. 7.

Table 3 summarizes the results from both the laboratory test and the in-situ sand well immersion test. It also includes data from similar tests at a nearby site about 800 m away, within the same geomorphological unit^33^. This allows for comparison under similar engineering-stratigraphic and geomorphological conditions, supporting comparability between the present and nearby sites. Table 3 shows a large difference between laboratory and in-situ sand well immersion results. These two tests lead to opposing conclusions about collapsibility type and grade. Laboratory tests at both sites show greater self-weight collapse settlement, while the immersion pit test shows heave instead of settlement. Laboratory results are similar for both sites, indicating self-weight collapsibility. In contrast, both in-situ tests (at this site and the adjacent site) find no self-weight collapsibility. This consistency in in-situ test results supports the reliability of using the sand well immersion test to evaluate deep loess collapsibility.

**Table 3:** Comparison of different evaluation methods for the loess collapsibility.

Test site	This test site		Adjacent test site	
Evaluation method	laboratory test	in-situ sand well immersion test	Laboratory test	Immersion pit test
Self-weight collapse Δ_ZS_/mm	176 ~ 255	6	216	9*
Total collapse/mm	199 ~ 309	28	/	/
Collapsibility type	Self-weight	Non-self-weight	Self-weight	Non-self-weight
Collapsibility grade	Ⅱ(Moderate)	Ⅰ(Slight)	/	/
Correction coefficient $\beta_{0}{\prime}$	/	0.03	/	0

The symbol * in the table denotes upward. the nearby site reported in Ref.^33^ is approximately 800 m from the present test site and belongs to the same geomorphological unit.

To avoid disturbance of the natural infiltration field, all sampling boreholes were immediately backfilled and sealed after sampling using compacted loess and bentonite, thereby preventing the formation of artificial preferential vertical flow paths.

### Analysis of the discrepancy between laboratory test and in-situ sand well immersion test

According to the code for building construction in collapsible loess regions, two primary methods are used to assess loess collapsibility: laboratory compression tests and in-situ sand well immersion tests^15^. The laboratory compression test utilizes the layerwise summation method. The calculated values for site self-weight collapse settlement and total collapse settlement are obtained by applying corrections to values calculated from the measured self-weight collapsibility coefficient and the collapsibility coefficient. The in-situ immersion test directly provides the measured value of self-weight collapse deformation and the lower limit depth of self-weight collapse for the site’s loess. In-situ sand well immersion test investigates a larger volume of soil, effectively reflecting the influence of both micro- and macro- on soil properties. In-situ sand well immersion tests are considered good representations and reliable^34^.

In this study, discrepancies frequently occur between laboratory-calculated values and field-measured values for loess collapse. In fact, reports of significant differences between calculated self-weight collapse settlements obtained using standardized laboratory tests and measured field results have become increasingly common in recent years. According to Clause 4.4.3 of the Code for Building Construction in Collapsible Loess Regions (GB 50,025–2018), the calculated self-weight collapse settlement of a collapsible loess site is expressed as follows:

1 $$\Delta_{zs} = \beta_{0} \sum\limits_{i = 1}^{n} {\delta_{zsi} h_{i} }$$

In the formula: *Δ*_*ZS*_ is the calculated self-weight collapse settlement; *β*_0_ is the regional correction coefficient specified in the loess code; *δ*_zsi_ is the self-weight collapsibility coefficient of soil layer i; *h*_i_ is the thickness of soil layer *i*.

The calculated value of self-weight collapse settlement is primarily influenced by three factors: *β*_0_, *δ*_zsi_, and *h*_i_. Therefore, the following analysis of the discrepancy between the calculated and measured values focuses on these three factors.

- (1) Regional correction coefficient *β*_0_

The regional correction coefficient β₀ varies with regional soil conditions. The test site is in Zone III (the Guanzhong region), where β₀ is defined as 0.9^15^. However, numerous engineering cases indicate that significant differences in collapsibility can exist even in the Guanzhong loess zone^35^. In particular, the collapsibility characteristics of this site differ from those of the typical northern Guanzhong area. This distinction is primarily due to the following reasons. First, regarding hydrogeological conditions, water resources near the site are relatively abundant. Specifically, the Hao River and Jue River flow along the southern and northern sides of the site, respectively (Fig. 1). Both are perennial rivers located close to the test site, at approximately 2.0 km and 0.6 km, respectively. There is interaction between river water and groundwater. The groundwater table is about 33 m deep, with an annual fluctuation of approximately 3.0 m. Consequently, strata below 30 m are influenced by groundwater, leading to almost non-collapsibility. Second, the climatic environment contributes to these differences. The test site is located near the Qinling Mountains (Fig. 1). During the late Late Pleistocene, the site experienced a relatively humid sedimentary environment, influenced by the Qinling climatic zone, which contrasts with the dry conditions in the northern Guanzhong area.

③ Clay content. The test site’s loess shows a clay content of 44% (D < 0.005 mm), nearly double the Guanzhong regional average of 23%^35,36^. This high clay content likely reduces field collapsibility by limiting large pore connectivity and increasing adsorbed water and local swelling during wetting. Although the material and chemical environment differ from those of the present loess field test, swelling tests on GMZ24 bentonite under an Fe(II) reducing environment also indicate that clay-mineral-rich materials can exhibit notable swelling-related volume change upon hydration^37^. The literature reports similar findings for loess soils with elevated clay fractions, though actual behavior also depends on mineralogy, microstructure, and hydraulic pathways^29,38–40^. However, since mineral composition and specific surface area were not measured in this study, these mechanisms are offered as literature-based interpretations rather than verified conclusions. It is important to emphasize that mechanisms such as clay content and paleosol interlayers, while supported by previous studies, remain plausible explanations rather than definitive findings due to the absence of direct field microstructural or pore-pressure evidence. Pore Structure. Field inspection shows that the soil at this site has an embedded microporous, cemented structure, contrasting with the macroporous, semi-cemented framework seen at other sites.

Therefore, the loess collapsibility in the test site is markedly lower than that of northern Guanzhong. Different correction coefficients should be applied based on specific regional soil conditions, rather than adhering to a uniform coefficient prescribed by the code. This interpretation is also consistent with international studies showing that the engineering response of loess is strongly controlled by regional differences in clay fraction, mineralogy, microstructure, suction evolution, and wetting path, so direct transfer of a single coefficient across different loess settings may be inappropriate^16,29,38–41^.

Referring to the Standard for building construction in collapsible loess regions (GB 50,025–2018), the ratio of the measured self-weight collapse settlement to the calculated self-weight collapse settlement (without multiplying by *β*_0_), computed using Eq. (2), can serve as the regional correction coefficient *β*’ 0 for this area:

2 $$\beta_{0}{\prime} = \frac{{\Delta{\prime}_{zs} }}{{\sum\limits_{i = 1}^{n} {\delta_{zsi} h_{i} } }} = \frac{{\Delta{\prime}_{zs} \beta_{0} }}{{\Delta_{zs} }}$$

In the formula: *β*’ 0 is the recommended value of *β*_0_; Δ^’^_ZS_ is the measured value of *Δ*_ZS_. The explanations for the remaining symbols are the same as above.

The maximum measured self-weight collapse settlement, Δ’_ZS_ (6.0 mm), and the minimum calculated settlement, Δ_ZS_ (176 mm), are adopted to ensure engineering safety for this test site. The calculated *β*’ 0 is 0.03, as shown in Table 3.

It should be emphasized that this back-calculated *β*’ 0 value is derived primarily from the field measurements at the present site and should therefore be regarded as a preliminary site-specific engineering suggestion rather than a statistically universal regional coefficient. Although the nearby-site immersion evidence provides qualitative support for a similarly low field collapsibility response, it does not constitute a formal multi-site calibration.

For loess sites (accumulated wind-blown silt deposits) with similar geological settings, a practical evaluation procedure is recommended. Laboratory collapsibility tests—which measure how much soil compacts when wet—may first be used for preliminary screening. If the site involves deep or thick loess strata (layers of loess), deep groundwater, a special climatic background, high clay content, or a suspected discrepancy between laboratory prediction and actual field response, an in-situ immersion test (soaking the soil in place to assess real-world behavior) or sand well immersion test (introducing water into boreholes filled with sand to simulate wetting effects) should be conducted for verification. When laboratory and field results are inconsistent, the field response should be given priority in the final engineering classification. The back-calculated *β*’ 0 value (an engineering parameter reflecting soil collapse potential) should be used only as a supplementary reference for sites with similar stratigraphic, climatic, and hydrogeological conditions.

- (2) Self-weight collapsibility coefficient *δ*_zsi_

The discrepancy between the calculated and measured self-weight collapse settlements is related to the testing process for determining the self-weight collapsibility coefficient *δ*_zsi_: ① Disturbance to soil samples during laboratory testing. The processes of exploratory well sampling, transportation, and specimen preparation with a cutting ring inevitably disturb the samples. This Disturbance causes some damage and alteration to the soil structure, thereby affecting the determination of the collapsibility coefficient. ② Potentially lower degree of saturation achieved in field tests. In laboratory tests, the soil specimen is thin and has a small volume, which provides favorable conditions for drainage and gas expulsion. Consequently, the specimen can reach a relatively high degree of saturation quickly after immersion. In field tests, however, gas expulsion from the soil mass (especially in deep layers) must occur over a longer path. Some gases exist in occluded forms and are not in contact with the atmosphere. This makes gas expulsion more difficult in field tests compared to laboratory conditions, potentially resulting in a lower final degree of saturation upon immersion. ③ Differences in stress conditions. Laboratory tests are conducted under confined (oedometric) stress conditions. These confining stresses restrain lateral deformation of the loess, thereby enhancing its compressive structural strength and reducing self-weight collapse deformation associated with lateral swelling. This constitutes a one-dimensional deformation problem. In contrast, the in-situ sand well immersion test in this study is performed under conditions that allow lateral deformation. The water infiltration process involves not only an increase in compressive stress but also the development of shear stresses, representing a three-dimensional deformation problem. ④ Differences in the extent of water immersion. Laboratory tests essentially simulate the immersion of each soil layer over an effectively infinite horizontal area. They do not account for interactions between water and gas movement through soils with different stratigraphic structures. The in-situ sand well immersion test introduces water within a finite area, creating a defined saturation boundary. This method better simulates the actual scenario of localized water ingress into a foundation. Therefore, the in-situ sand well immersion test better aligns with real-world engineering conditions.

From a mechanistic perspective, wetting-induced collapse of loess is closely related to the destruction of its metastable microstructure^18,21,29^. Recent studies have shown that inundation can crush macropores, narrow pore throats, and transform unstable point-to-point contacts into more stable edge-type contacts, while particle disintegration, clay swelling, and partial dissolution of carbonate cement further weaken the structural skeleton of the loess^18,21,29^. In addition, hydro-mechanical and stress-path studies indicate that wetting under constant load is essentially a suction-reduction process; as suction decreases and saturation increases, the effective stress state and hydraulic properties evolve markedly, and under saturated or contractive conditions, positive pore-water pressure may also develop, further reducing soil strength^29–32^. Although pore-water pressure and stress path were not directly monitored in the present study, these reported mechanisms help explain why the field collapse response depends not only on the laboratory collapsibility coefficient, but also on the actual saturation path, drainage and gas-expulsion conditions, and three-dimensional stress redistribution during in-situ immersion^34–36^.

- (3) Soil layer thickness *h*_*i*_

The value of hi is determined by the sampling interval. The code requires collecting one soil sample every 1–2 m to test its self-weight collapsibility coefficient, which is then used to calculate the self-weight collapse settlement. In this method, each soil sample corresponds to a soil layer thickness of 1–2 m^15^. However, loess deposits often comprise intermixed soil units with differing degrees of collapsibility. Therefore, increasing the sampling interval increases hi, which reduces the representativeness of the samples. Conversely, a smaller sampling interval improves sample representativeness. For instance, in the laboratory tests of this study, the sampling interval for borehole SJ03 was 1 m. According to current loess code, the calculated self-weight collapse settlement for borehole SJ03 is 210 mm. For boreholes TJ02 and TJ10, samples were collected at 1 m intervals for strata above 10 m depth and at 2 m intervals for strata below 10 m, resulting in calculated settlements of 255 mm and 176 mm, respectively. These results demonstrate that variations in sampling intervals produce distinct calculated self-weight collapse settlements for the loess stratum, underscoring the significant effect of soil layer thickness hi on the calculated value (Table 4).

**Table 4:** Summary of the main discrepancies between laboratory and in-situ collapsibility evaluations.

Factor category	Source of discrepancy	Evidence/basis in this study	Engineering implication
Calculation parameter	Regional correction coefficient *β*_0_	The code-specified value for Zone III is *β*_0_ = 0.9, whereas the back-calculated value from the present field case is *β*’ 0 = 0.03	The code-specified coefficient may overestimate field collapsibility at this site; the back-calculated value should be treated only as a site-specific supplementary reference
Site condition	Hydrogeological and climatic background	The test site has a relatively high groundwater table, is close to perennial rivers, and experienced a more humid paleo-environment than the typical northern Guanzhong area	A uniform regional coefficient may be unsuitable for loess sites with similar hydrogeological and climatic conditions
Soil material/structure	Clay content and pore structure	The clay content at the test site is 44%, and the soil is characterized by an embedded microporous, cemented structure	High clay content and microporous structure may contribute to lower field collapsibility than that predicted by laboratory testing
Testing condition	Stress state and deformation boundary	Laboratory tests are essentially one-dimensional and confined, whereas the in-situ sand well immersion test involves three-dimensional wetting and deformation under a finite boundary	In-situ testing may better represent the actual wetting and deformation conditions of real foundations
Saturation path/drainage condition	Gas expulsion and degree of saturation	Laboratory specimens can saturate relatively rapidly, whereas deep field loess may retain occluded gas and follow a different saturation path	Laboratory tests may overestimate collapse when rapid and nearly complete saturation is implicitly assumed
Sampling representativeness	Sample disturbance and layer-thickness representation (*h*_i_)	Different sampling intervals among boreholes produced different calculated self-weight collapse settlements, reflecting the influence of layer representation in the calculation	Laboratory-based evaluation is sensitive to sample representativeness and discretization of soil layers

Interpretations related to clay effects, local swelling, and saturation response are supported by field observations and previous literature, but were not directly verified in the present study by dedicated microstructural, pore-water-pressure, stress-path, or swelling tests.

## Conclusions

This study focuses on the Shenheyuan loess. We monitored the water content and collapse settlement response of the loess stratum with the in-situ sand well immersion test. By comparing these results with the laboratory collapse test, we draw the following conclusions:

1. For the Shenheyuan loess stratum, results from the in-situ sand well immersion test show no significant collapse settlement in the loess layers. The maximum settlement is less than 70 mm, below the criterion for self-weight collapsibility. Therefore, the site in southern Guanzhong is classified as non-self-weight-collapsible.
2. There is a significant discrepancy between the laboratory tests and the in-situ sand well immersion test. The laboratory tests classified the site as self-weight collapsible with grade II collapsibility. However, the in-situ test results differ. A comparative analysis from an adjacent site shows that the in-situ test provides a more accurate assessment of loess collapsibility.
3. The main reason for the difference between field-measured and laboratory results is the regional correction coefficient in the code. This coefficient does not consider the site’s unique hydrogeological conditions, climate, and soil structure. Mechanistic interpretations, such as the roles of clay content and paleosols, have support in the literature. They remain plausible explanations rather than direct findings. Thus, using code-provided values directly may lead to misjudgment of collapsibility.

## Data Availability

The datasets generated and/or analyzed during the current study are available from the corresponding author, Dr. Zhang, upon reasonable request. Requests for data should be addressed to Dr. Zhang at zhangming@zua.edu.cn.
